# Complete Genome Sequence of an *Escherichia coli* O121:H19 Strain from an Outbreak in Canada Associated with Flour

**DOI:** 10.1128/genomeA.01561-17

**Published:** 2018-01-25

**Authors:** James Robertson, Janet Lin, Paul N. Levett, Celine Nadon, John Nash, Chrystal Berry

**Affiliations:** a Division of Enteric Diseases, National Microbiology Laboratory, Public Health Agency of Canada, Guelph, Ontario, Canada; b Saskatchewan Disease Control Laboratory, Regina, Saskatchewan, Canada; c Division of Enteric Diseases, National Microbiology Laboratory, Public Health Agency of Canada, Winnipeg, Manitoba, Canada

## Abstract

Here, we present the first complete genome sequence of an *Escherichia coli* non-O157 Shiga-toxin producing isolate, 16-9255, from serotype O121:H19. This strain is notable as a clinical case recovered from a recent Canadian flour-associated outbreak event.

## GENOME ANNOUNCEMENT

Between December 2016 and May 2017, 29 clinical cases of *Escherichia coli* O121:H19 were reported to Canada’s foodborne surveillance and outbreak response network, PulseNet Canada, as part of a national outbreak investigation linking these cases to the consumption of contaminated flour products. We present here the complete genome sequence of isolate 16-9255, recovered from an outbreak clinical case and selected for genome closure, as it displays the predominant laboratory subtyping patterns included in the outbreak case definition.

Genomic DNA was isolated from overnight culture using the automated Qiagen EZ1 DNA tissue extraction kit. Paired-end sequencing (2 × 300 bp) of a 16-9255 Nextera XT genomic DNA library was performed on an Illumina MiSeq platform. The Oxford Nanopore Technology (ONT) MinION sequencer was employed to produce long reads enabling genome closure. ONT libraries were prepared using the rapid sequencing kit (RAD-0002) and an R9.4 flow cell. Sequencing was performed for 48 h, and 0.84 Gb of sequencing data was produced, representing 75,576 reads, with an average read length of 11 kb and longest read length of 310 kb. Hybrid assemblies were produced using Unicycler version 0.4.3 (1), yielding two circular contigs representing the chromosome (5,397,251 bp) and a plasmid (81,950 bp). Annotation with Prokka version 1.12 (2) predicted genes for 5,330 protein-coding sequences, 22 rRNAs, 102 tRNAs, 1 transfer-messenger RNA (tmRNA) gene, and 2 clustered regularly interspaced short palindromic repeat (CRISPR) systems. Additional predicted genes include that encoding the multidrug efflux pump Mdt, a *mar* locus encoding multiple antibiotic resistance, and genes encoding resistance to copper, tellurite, and quaternary ammonium compounds. A 45.8-kb Shiga toxin 2a-producing lambdoid phage was identified with PHASTER (3, 4). Similar to other reported *E. coli* O121:H19 strains, 16-9255 carries a 39-kb locus of enterocyte effacement (LEE) harboring several genes whose products include intimin, a type III secretion system apparatus, secreted effectors, and regulators of the LEE. Together, the products of the LEE are responsible for causing the attaching and effacing lesions characteristic of enterohemorrhagic Shiga toxin-producing *E. coli* (STEC) strains. Prokka annotation also identified the plasmid-carried virulence genes *ehxA*, *toxB*, and *espP* encoding the virulence-associated enterohemolysin, toxin B, and an extracellular serine protease, respectively.

This sequence provides a high-quality reference for genome assemblies of *E. coli* O121 strains and has relevant application to other *E. coli* O121 and non-O157 STEC genomic studies.

### Accession number(s).

The complete genome sequence of isolate 16-9255 was deposited in the NCBI GenBank database under the accession number CP022407.
